# The SLC transporter in nutrient and metabolic sensing, regulation, and drug development

**DOI:** 10.1093/jmcb/mjy052

**Published:** 2018-09-18

**Authors:** Yong Zhang, Yuping Zhang, Kun Sun, Ziyi Meng, Ligong Chen

**Affiliations:** 1 School of Pharmaceutical Sciences, Tsinghua University, Beijing, China; 2 Advanced Biotechnology and Application Research Center, School of Chemistry and Biological Engineering, University of Science and Technology Beijing, Beijing, China

**Keywords:** SLC transporters, disease loci, nutrient and metabolic sensing, drug development

## Abstract

The prevalence of metabolic diseases is growing worldwide. Accumulating evidence suggests that solute carrier (SLC) transporters contribute to the etiology of various metabolic diseases. Consistent with metabolic characteristics, the top five organs in which SLC transporters are highly expressed are the kidney, brain, liver, gut, and heart. We aim to understand the molecular mechanisms of important SLC transporter-mediated physiological processes and their potentials as drug targets. SLC transporters serve as ‘metabolic gate’ of cells and mediate the transport of a wide range of essential nutrients and metabolites such as glucose, amino acids, vitamins, neurotransmitters, and inorganic/metal ions. Gene-modified animal models have demonstrated that SLC transporters participate in many important physiological functions including nutrient supply, metabolic transformation, energy homeostasis, tissue development, oxidative stress, host defense, and neurological regulation. Furthermore, the human genomic studies have identified that SLC transporters are susceptible or causative genes in various diseases like cancer, metabolic disease, cardiovascular disease, immunological disorders, and neurological dysfunction. Importantly, a number of SLC transporters have been successfully targeted for drug developments. This review will focus on the current understanding of SLCs in regulating physiology, nutrient sensing and uptake, and risk of diseases.

## Introduction

A complex system exists for maintaining human health that consists of dietary components, environmental chemicals, and pharmaceuticals that interact with genes for normal activity. Although there has been much research that has been performed regarding the various nutrients and metabolites required for health, considerably less focus has been placed on their transport in the body. This is partly because of technical challenges and the priority for noted genes. Thus, the identification of transporters has lagged behind other studies. Membrane transporters mainly include members of the ion and water channels, which are ATP-binding cassette (ABC) and solute carrier (SLC) transporters.

**Figure 1:**
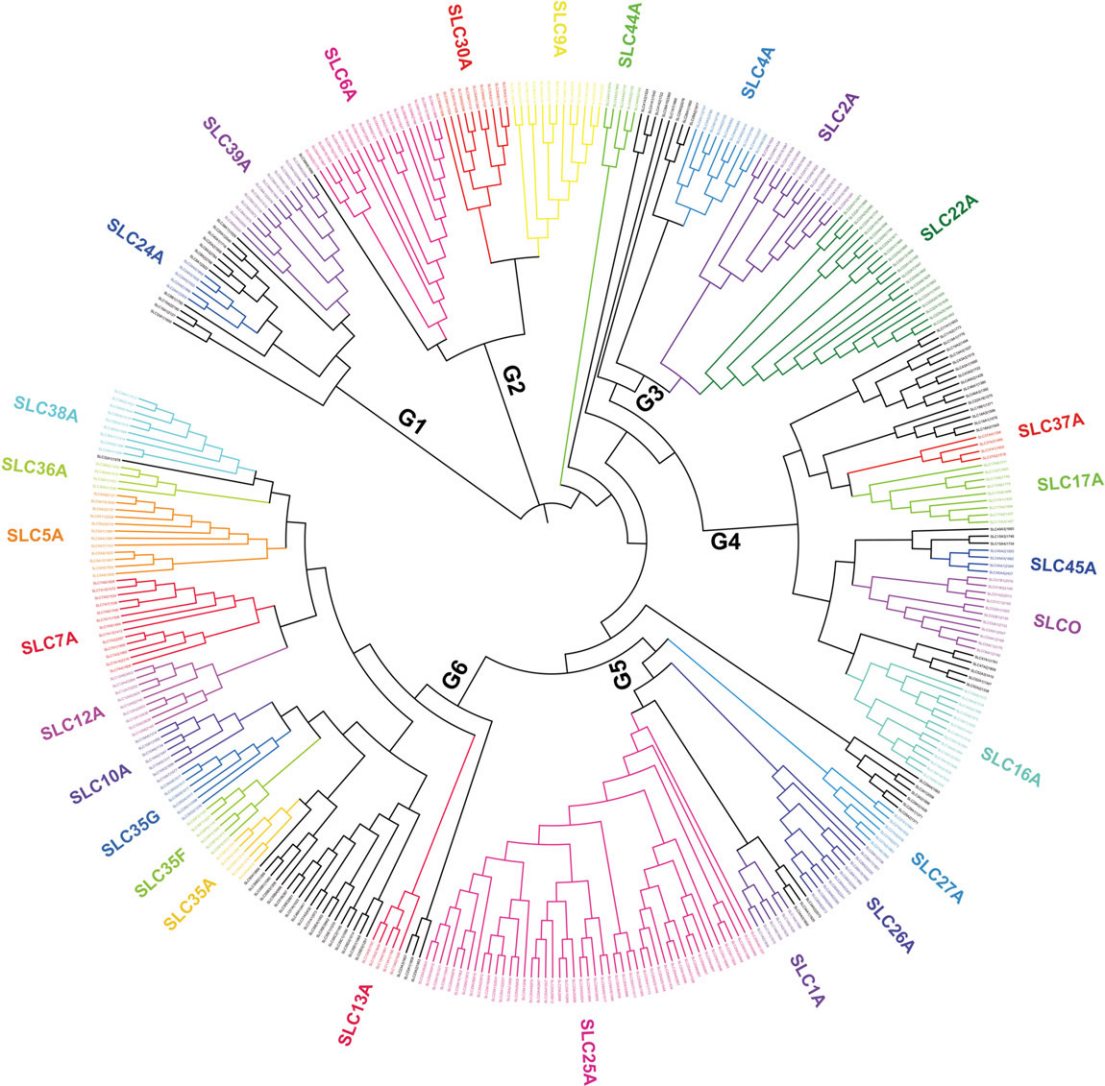
The phylogenetic tree of SLC superfamily. Different colors represent different subfamilies (see Supplementary Methods section). The SLCs are mainly classified into six groups: (i) Group 1 includes the SLC8, SLC24, and SLC39 families; (ii) Group 2 includes the SLC6, SLC9, and SLC30 families; (iii) Group 3 includes the SLC2, SLC4, SLC22, SLC28, and SLC41 families; (iv) Group 4 includes the SLC16, SLC17, SLC18, SLC37, SLC43, SLC45, and SLC47 families; (v) Group 5 includes the SLC1, SLC25, SLC26, SLC27, SLC29, and SLC34 families; (vi) Group 6 includes the SLC5, SLC7, SLC10, SLC12, SLC13, SLC23, SLC35, SLC36, and SLC38 families. Subfamily similarities may imply common ancestry and suggest possible functional similarity.

Current research has focused on the roles of SLC transporters in metabolic diseases. Human SLC transporter families contain 400 genes and 52 subfamilies. We describe a comprehensive similarity relationship of SLCs superfamily (Figure 1). SLCs contribute to the transmembrane transport of various substrates such as inorganic ions, amino acids, fatty acids, neurotransmitters, and saccharides. Many SLC susceptibility loci have been strongly associated with metabolic diseases including insulin resistance, type 2 diabetes mellitus (T2DM), elevated blood pressure, chronic kidney disease (CKD), gout, asthma, inflammatory bowel disease (IBD), cancer, dementia, and anxiety disorders (Table 1).

**Table 1:** Representative SLC transporter-relevant human diseases.

SLC	Human diseases	Known substrates	References
SLC2A2	T2DM insulin resistance	Facilitated glucose transporter	Dupuis et al. (2010)
SLC16A11	T2DM	Transport of pyruvate across the plasma membrane	Rusu et al. (2017)
SLC30A8	T2DM Insulin resistance	Zinc transporter 8	Dupuis et al. (2010)
SLC6A1	Anxiety disorders	GABA transporter	Thoeringer et al. (2009)
SLC6A12	Schizophrenia in a Korean population	GABA transporter	Park et al. (2011)
SLC6A15	Depression	Branched-chain amino acids, particularly leucine, valine, isoleucine, and methionine	Hyde et al. (2016)
SLC30A10	Neurologic, hepatic, and hematologic disturbances	Manganese transport	Quadri et al. (2012)
SLC24A4	Alzheimer’s disease	Calcium transport	Lambert et al. (2013)
SLC2A9	Gout	Urate	Dehghan et al. (2008); Kolz et al. (2009)
SLC16A9	Gout	Urate	Kolz et al. (2009); Yang et al. (2010)
SLC17A1	Gout	Sodium-dependent phosphate transporter 1; a renal transporter of uric acid	Kolz et al. (2009); Hollis-Moffatt et al. (2012); Nakayama et al. (2017)
SLC17A3	Gout	Urate	Dehghan et al. (2008)
SLC22A11	Gout	Organic anion transporter 4	Yang et al. (2010); Flynn et al. (2013)
SLC22A12	Gout	Urate transporter 1	Kolz et al. (2009); Flynn et al. (2013); Nakayama et al. (2017)
SLC4A7	Elevated blood pressure	Electroneutral Na^+^/HCO_3_^−^ cotransporter NBCn1	Lu et al. (2015); Ehret et al. (2016); Wang et al. (2017); Ng et al. (2017)
SLC6A13	Elevated blood pressure chronic kidney disease (CKD)	GABA transporter	Levy et al. (2009); Kottgen et al. (2010); Liu et al. (2011)
SLC8A1	Elevated blood pressure	Sodium(Na^+^)-calcium(Ca^2+^) exchanger 1	Warren et al. (2017)
SLC12A1	Blood pressure variation	Kidney-specific sodium–potassium–chloride cotransporter; accounts for most of the NaCl resorption	Ji et al. (2008)
SLC12A3	Blood pressure variation	Renal thiazide-sensitive sodium-chloride cotransporter	Ji et al. (2008)
SLC14A2	Elevated blood pressure	Urea transporter	Warren et al. (2017)
SLC22A4/5	Elevated blood pressure	Gothioneine and carnitine	Wain et al. (2017)
SLC24A3	Elevated blood pressure	K^+^-dependent Na^+^/Ca^2+^ exchanger 3	Warren et al. (2017)
SLC35F1	Elevated blood pressure	Unknown	Warren et al. (2017)
SLC39A8	Elevated blood pressure	Zinc transport	Ehret et al. (2016)
SLC39A13	Elevated blood pressure	Zinc transport	Ehret et al. (2016)
SLC25A32	Blood pressure	Unknown	Fowdar et al. (2017)
SLC7A9	CKD	Transport of cystine and neutral and dibasic amino acids	Kottgen et al. (2010)
SLC34A1	CKD	Sodium–phosphate cotransporter	Kottgen et al. (2010)
SLC22A2	CKD	Metformin, cisplatin, and lamivudine	Kottgen et al. (2010); Liu et al. (2011)
SLC22A5	Asthma	Carnitine transporter	Torgerson et al. (2011); Moffatt et al. (2010)
SLC30A8	Asthma	Zinc transporter 8	Himes et al. (2012)
SLC22A23	Bronchodilator responsiveness in asthma	Unknown	Noguchi et al. (2011)
SLC25A15	Bronchodilator responsiveness in asthma	Unknown	Drake et al. (2014)

For the human SLC family members, Table 1 summarizes their types of predominant transport substrates and links to common diseases (T2DM, depression, Alzheimer’s disease, gout, elevated blood pressure, CKD, and asthma).

**Figure 2:**
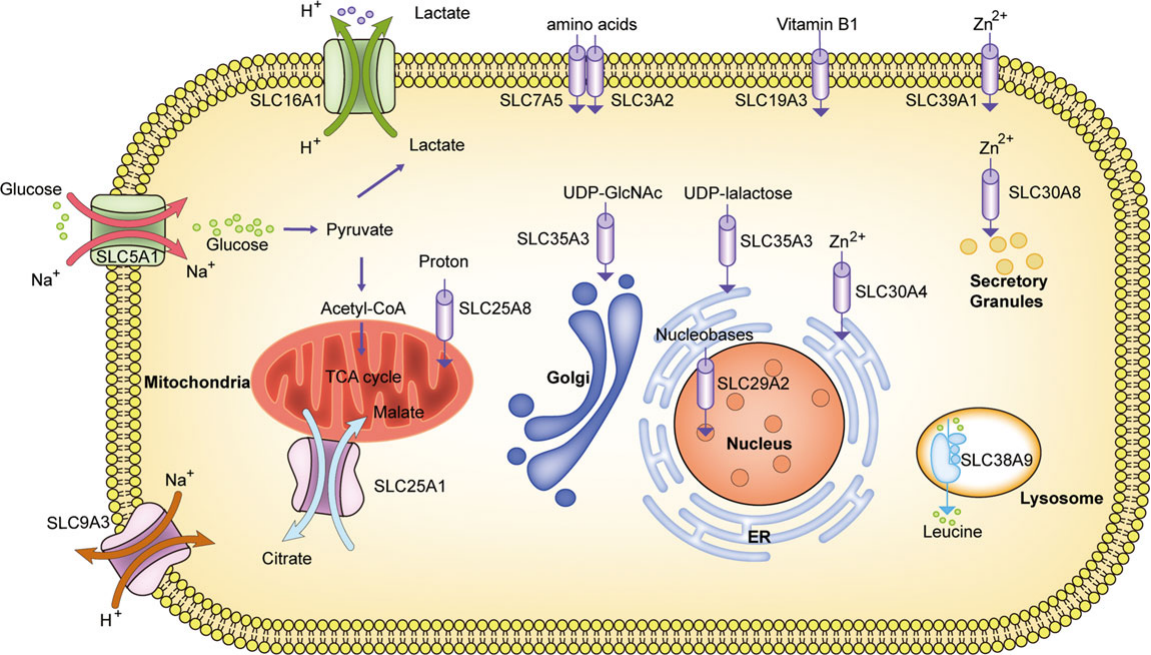
Representative modes of SLC transport. SLCs constitute a dynamic work coordination for living cells. Different modes of SLC transport, including cotransporters, exchangers, facilitated and orphan transporters, are marked by different shapes and colors. The representative SLC transporters include SLC3A2/SLC7A5 (amino acids), SLC5A1 (glucose and Na^+^), SLC9A3 (Na^+^/H^+^ exchanger), SLC16A1 (lactate), SLC19A3 (thiamine), SLC25A1(citrate/malate exchanger), SLC25A8 (protons), SLC29A2 (nucleobases), SLC30A4 (Zn^2+^ to ER), SLC30A8 (Zn^2+^ to granules), SLC35A3 (UDP-GlcNAc to Golgi), SLC35A3 (UDP-galactose to ER), SLC38A9 (leucine), and SLC39A1 (Zn^2+^ to intracellular fluid). SLCs participate in important biological functions for glycolysis, acidification, TCA cycle, and nutrient supply. Among these, SLC3A2 and SLC7A5, considered as heteromeric amino acid transporters, are collaborative for amino acid transport. The activities of SLCs cover all organelles from nucleus to cell membrane.

The transmembrane transport by SLCs could be mainly divided into four modes of transport, including cotransporter, exchanger, facilitated transporter, and orphan transporter (Figure 2). Cotransporter is defined as the movement of one substrate via concentration gradient coupled with other components. The first discovered cotransporter was SLC5A1, which translocates Na^+^ and glucose in both directions across the epithelial cell membrane and thus provides glucose for glycolysis. Another Na^+^/glucose cotransporter for glucose reabsorption is SLC5A2, which mediates sodium uptake and glucose reabsorption in the proximal tubular cells (Gerich, 2010). In the second class, exchanger is apparently referred to two substrates across the membrane in opposing direction. It was found that SLC9A3 mediates the translocation of Na^+^ and H^+^ in either direction across the membrane for preventing acidification in the gut. This mechanism is called the Na^+^/H^+^ exchanger. An additional important transporter in control of cell acidification is SLC16A1, which mediates the outward transport of lactate and H^+^ from glycolysis. In addition, citrate is exchanged with H^+^/malate on the inner mitochondrial membrane during the TCA cycle by SLC25A1. The third class is the facilitated transporter, which is considered as the spontaneous passive transport of one substrate without coupled components. For example, SLC25A8 facilitates the transfer of H^+^ from the inner to the outer mitochondrial membrane for energy metabolism. Another facilitating transporter is SLC19A3, which contributes to the intestinal absorption of thiamine uptake. Other complex transporters were called orphan transporter, and its substrates and consequent functions remain to be identified. The most recently found transporting mode is that of SLC38A9, which translocates leucine from the lysosome to the cytoplasm when mTORC1 binds to the lysosomal membrane. Besides, a series of drugs also can be transported by SLCs (Figure 2).

**Figure 3:**
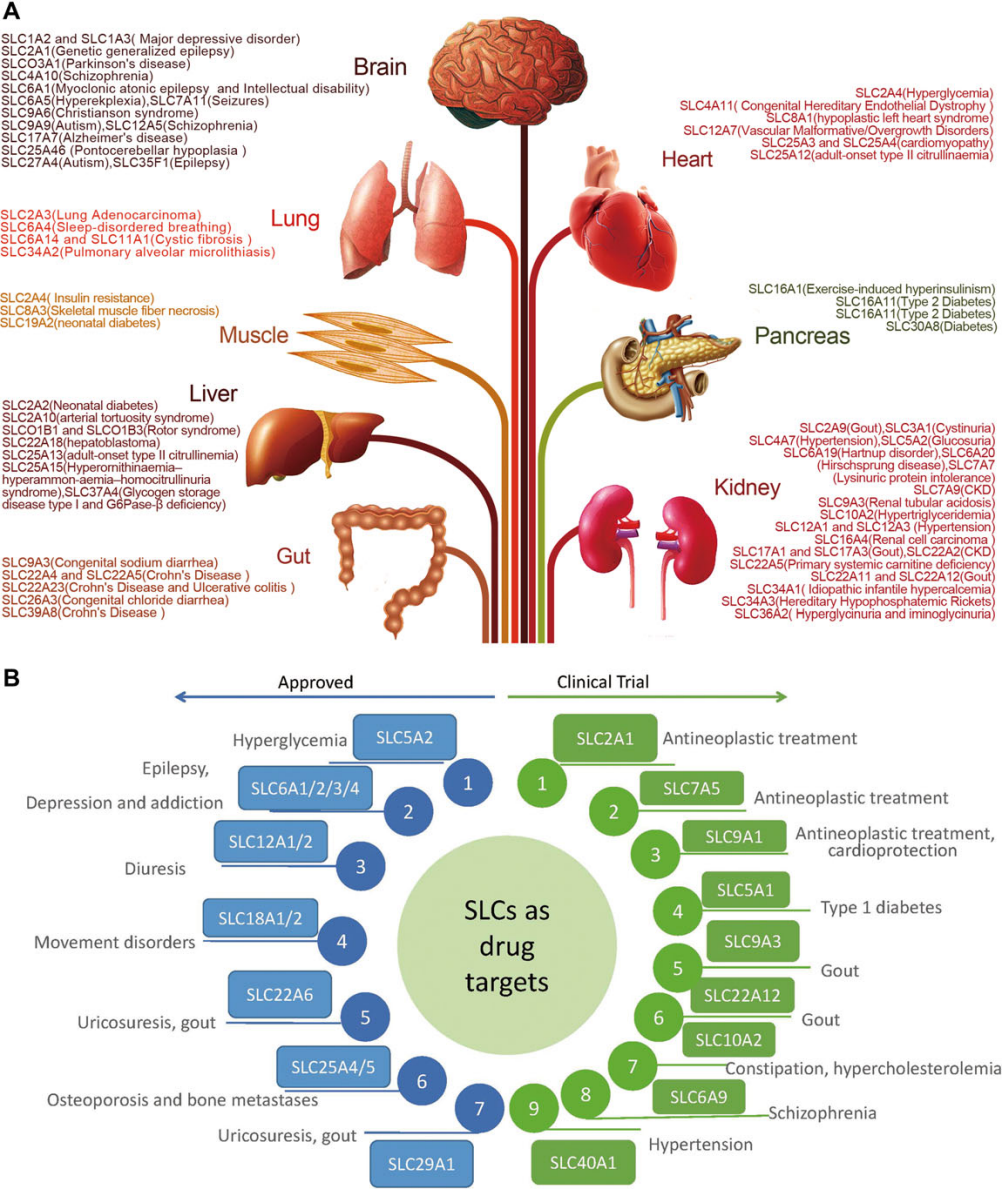
(**A**) Tissues and diseases associated with SLCs. Major advances in understanding of the relationship between disease susceptibility and SLCs have been made. Accumulation of gene mutations and GWAS studies have demonstrated that SLCs play a crucial role in human diseases. SLCs are specifically expressed in different organs and involved in the pathogenesis of various human diseases. SLC members in the same family have been described that differ in the organ expression with different functions. The brain and kidney are two target organs for most high expression of SLCs-mediated diseases. Thus, SLCs are promising for neurologic and metabolic target. (**B**) SLC inhibitors for drug targets. The current SLC drug development is promising. Previous approved drugs were widely used for treatment of hyperglycemia, diuresis, movement disorders, uricosuresis, gout, and so on. Newly testing SLC drugs have the potential to exert antineoplastic effects, ameliorate Type 1 diabetes, resist constipation, protect from hypertension and schizophrenia. Data were cited from Rask-Andersen et al. (2013) and Cesar-Razquin et al. (2015).

Except for different modes, different organelles are also crucial for SLCs. For example, the SLC30A family mediates the transport of zinc in the nucleus (SLC30A9), endosome (SLC30A4), Golgi (SLC30A5, SLC30A6, SLC30A7), and secretory granules (SLC30A8) for alkaline phosphatases (ALPs). The SLCs are finely cooperative to regulate substrate movement. Moreover, SLCs regulate tissue-specific substrate movement associated with diseases (Figure 3A). Here, we review the characteristics and functional roles of various SLCs.

## SLCs in human diseases

As the development of sequencing technology, the contributions of genetic variants to common diseases become more clear. To explore the functions of SLC transporters, the strong associations between SLCs and a variety of diseases are first established (summarized in Table 1).

### SLCs for insulin resistance and T2DM

Obesity is a driver of complications such as insulin resistance and T2DM, which are considered to be a global burden (Zheng et al., 2018). Although the etiology of T2DM is partially understood, new genetic loci continuously emerge. SLC2A2 was previously observed in a GWAS study for the loci of insulin resistance and T2DM (Dupuis et al., 2010). However, SLC2A2 is not the principal glucose transporter in human pancreatic β cells (McCulloch et al., 2011). SLC2A2, which also functions as the main liver glucose transporter, has been found to transport glucosamine at high affinity and other glucose and fructose molecules with lower affinity (Uldry et al., 2002). It has been demonstrated that glucosamine inhibits β-cell glucokinase activity and reduces the insulin response (Yoshikawa et al., 2002). In addition, glucosamine can also induce endoplasmic reticulum (ER) stress in monocytes and hepatocytes involved in accelerating atherosclerosis in hyperglycemia (Werstuck et al., 2006).

SLC30A8, also known as zinc transporter 8 (ZnT8), is highly expressed in the pancreas that supports a potential role for affecting the endocrine system (Huang and Tepaamorndech, 2013). A GWAS study revealed that SLC30A8 is another common SLC loci for T2DM, and its polymorphisms are linked to the T2DM susceptibility of various populations (Tabara et al., 2009; Dupuis et al., 2010; Seman et al., 2015; Phani et al., 2016; Nikitin et al., 2017). Although ZnT8-null mice have a moderate glucose tolerance (Nicolson et al., 2009), glucose-stimulated insulin secretion was abolished in Slc30a7 and Slc30a8 double knockout mice (Syring et al., 2016). More recently, it was shown that selective deletion of ZnT8 in pancreatic β-cells induced a significant impairment in glucose tolerance, while ZnT8 overexpression in β-cells resulted in significant improvements in glucose tolerance (Mitchell et al., 2016). Interestingly, human Slc30a8 transgenic mice exhibited a decrease in pancreatic [Zn^2+^] concentration and higher insulin and glucose tolerance after consuming a high-fat diet (Li et al., 2017).

It has been demonstrated that SLC16A11 is a locus associated with T2DM in the Mexico population (SIGMA Type 2 Diabetes Consortium et al., 2014; Miranda-Lora et al., 2017). More recently, it was found that two distinct mechanisms of T2DM are a decrease in liver SLC16A11 expression and disruption of basigin (BSG) (Rusu et al., 2017). However, the precise mechanisms of SLC16A11 leading to T2DM pathogenesis in humans remain unclear and further studies are required.

### SLCs for elevated blood pressure

Elevated blood pressure continues to be a global public health problem, and it is also a major contributor to coronary heart disease (Benjamin et al., 2017). Thus far, over 200 genetic loci for blood pressure have been identified in GWAS studies, and the SLCs are involved including SLC4A7, SLC6A13, SLC8A1, SLC12A3, SLC25A32, SLC39A8, and SLC39A14 (Ji et al., 2008; Ehret et al., 2016; Fowdar et al., 2017; Wain et al., 2017; Wang et al., 2017; Warren et al., 2017).

High sodium intake is one of the major risk factors for elevated blood pressure (He and MacGregor, 2011). Thus, Na^+^ transporters may play an important role in blood pressure variation. Recent evidence has revealed that SLC4A4 but not SLC4A7 was associated with dietary sodium intake-induced blood pressure responses in the Han Chinese population (Guo et al., 2016).

Generally, intracellular pH affects intracellular enzymatic activities and contributes to vascular function (Boedtkjer et al., 2006). The SLC4A7 gene is a Na^+^/HCO_3_^−^ transporter that mediates intracellular pH regulation in smooth muscle cells from the heart (Boedtkjer et al., 2011). Although SLC4A7-deficient mice displayed a lower pH in mesenteric artery smooth muscle and endothelial cells, they exhibited a modest hypertensive state (Boedtkjer et al., 2016). More recently, it was discovered that SLC4A7-deficient mice affect the migration of vascular smooth muscle cells and remodeling of carotid arteries that might be involved in the pathogenesis of occlusive artery disease (Ng et al., 2017).

### SLCs for CKD

The incidence and prevalence of CKD are increasing worldwide. Previous GWAS studies have identified SLC6A13, SLC7A9, and SLC22A2 as susceptibility genes of CKD (Kottgen et al., 2010; Liu et al., 2011). SLC6A13, a GABA transporter, has been a susceptibility gene for both hypertension and CKD (Kottgen et al., 2010).

SLC22A2 belongs to the organic cation transporter (OCT) family (Fujita et al., 2006). SLC22A2 mRNA is abundant in the kidney, especially the basolateral membrane of the proximal tubules, pointing to a role in the control of kidney uptake and nephrotoxicity (Ji et al., 2002). In early studies, SLC22A2 expression was significantly lowered in adenine-induced chronic renal failure rats (Nishihara et al., 2007). Moreover, cisplatin-induced kidney damage was abolished by SLC22A2 absence via p53 signaling (Sprowl et al., 2014). It is also evident that SLC22A2 polymorphisms were associated with reduced cisplatin-induced nephrotoxicity and maintenance of the estimated glomerular filtration rate in patients (Chang et al., 2017).

### SLCs for gout

Gout is characterized by chronic hyperuricemia and the formation of monosodium urate monohydrate crystals around joints (Liebman et al., 2007). It is notable that uric acid transport seems to play a key role in the pathogenesis of gout. According to GWAS studies, SLCs including SLC2A9, SLC16A9, SLC17A1, SLC17A3, SLC22A11, and SLC22A12 were risk loci for gout (Dehghan et al., 2008; Kolz et al., 2009; Flynn et al., 2013). Because of different mechanisms, gout has been divided into renal overload gout and renal underexcretion gout. Human data showed that 30%–40% of urate is cleared in the intestine and most of the rest via the kidney (So and Thorens, 2010). However, whether the uric acid concentrations in the gut and kidney are regulated by different SLCs is not clear.

SLC2A9, which is mainly expressed in the liver, kidney, and intestine (jejunum), is the most common cause of hyperuricemia (DeBosch et al., 2014). A previous study showed that SLC2A9 participates in the reabsorption of filtered urate by the proximal tubules. In SLC2A9 knockout mice, the animals exhibited a complex metabolic syndrome of hyperuricemia, hyperuricosuria, spontaneous hypertension, and dyslipidemia (DeBosch et al., 2014).

SLC16A9 also mediates urate transport. The physiological roles of SLC16A9 in gout are related to intestinal urate clearance rather than decreased renal urate excretion (Nakayama et al., 2013). There are no data in the literature describing SLC16A9 deficiency. SLC17A1, also called sodium phosphate transport protein 1 (NPT1), is expressed on the apical membrane of renal tubular cells and liver cells. SLC22A12 was also expressed on the apical side of the renal proximal tubule. SLC22A12 knockout mice exhibited a higher ratio of urinary urate/creatinine excretion, suggesting that SLC22A12 deficiency leads to reduced renal reabsorption of urate (Hosoyamada et al., 2010).

### SLCs for neurological diseases and mental disorders

There have been rising rates of anxiety that require lifetime medical care, which have driven increased concerns (American Psychological Association, 2013). GWAS studies have implicated SLC3A1 and SLC6A15 in association with the increased risk of anxiety disorders and major depression (MD), respectively (Kohli et al., 2011; de Moor et al., 2015; Genetics of Personality Consortium et al., 2015; Otowa et al., 2016). SLC3A1 and SLC6A15 are amino acid transporters, and their substrate transport mechanisms remain unclear. A further attempt in animal revealed that SLC3A1 deficiency causes cystinuria, and its relation to anxiety requires further research (Saravakos et al., 2014; Mizukami et al., 2015). Consistent with human data, SLC6A15 knockout mice exhibited less anxiety- and depressive-like behavior in response to chronic social stress compared to wild-type mice, while SLC6A15 overexpression mice possessed increased anxiety-like behavior (Santarelli et al., 2016).

Alzheimer’s disease (AD) with progressive cognitive deterioration has quickly become widespread that affects everyday activities (Alzheimer’s Association, 2016). A GWAS meta-analysis determined that SLC24A4 was a susceptibility loci of AD (Lambert et al., 2013). SLC24A4, highly expressed in the brain and olfactory neurons, exchanged four Na^+^ for one Ca^2+^ and one K^+^ across the cell membrane via an electrochemical gradient (Schnetkamp, 2013). In animals, SLC24A4 deficiency displayed stronger olfactory neuronal adaptation and a prolonged termination phase in olfactory response (Stephan et al., 2011). Interestingly, SLC24A4 loss has no influence on the response sensitivity of odorant exposure in mice, indicating that other components in the complex olfactory system regulate sensory stimuli. More recently, it was demonstrated that olfactory action was altered by a loss of both SLC24A4 and CNGB1 in mice, which displayed markedly reduced EOG amplitude and change of resting sensitivity (Ferguson and Zhao, 2017).

### SLCs in immunological dysfunction

Asthma, characterized by airway lymphocyte infiltration and inflammation, is the most common chronic T helper (Th) type 2-related immunological childhood disorder (Boulet, 2018). The prevalence of allergies and asthma is continuously increasing, particularly in developed countries (Beghe et al., 2017). Two large-scale GWAS for asthma found that SLC22A5 and SLC30A8 were significantly associated with asthma (Moffatt et al., 2010; Noguchi et al., 2011). It has been confirmed that prednisolone (a drug commonly for asthma) with L-carnitine ester stimulate prednisolone absorption across the bronchial epithelial cells via a SLC22A5 mechanism (Mo et al., 2011). Moreover, it has been demonstrated that SLC22A23 and SLC25A15 are strongly associated with the bronchodilator responsiveness of asthma (Himes et al., 2012; Drake et al., 2014).

IBD, including Crohn’s disease and ulcerative colitis, also display an immunological dysfunction (Xavier and Podolsky, 2007). SLC transporters identified in current GWAS of IBD contain SLC2A4, SLC9A3, SLC9A4, SLC11A1, SLC22A4, SLC22A5, SLC25A15, SLC26A3, SLC30A8, SLC22A23, and SLC39A8 (Khor et al., 2011; Yamazaki et al., 2013; Liu et al., 2015). This suggests that glucose, iron, zinc, carnitine, chloride ion, and the transport of other unknown substrates are involved in complex mechanisms of IBD. The function of several functional genes has been successfully identified in an animal model. For example, Slc22a5-deficient mice develop spontaneous intestinal ulcers and perforation with intense lymphocytic and macrophage infiltration (Shekhawat et al., 2007).

### SLCs in cancer

Butyrate is a tumor suppressor that acted as a histone deacetylase inhibitor (Davie, 2003). SLC5A8 may contribute to tumor progression because its one important transport substrate is butyrate (Gupta et al., 2006). Moreover, SLC5A8 also participated in colonic lactate transport, and Slc5a8-deficient mice displayed lactaturia (Frank et al., 2008). There is decreased expression of SLC5A8 in most tumors due to the loss of lactate transport, which is a feature of the Warburg effect (Vander Heiden et al., 2009). The Warburg effect requires an increased glucose uptake for cancer cells, which aggravates the expression of glucose transporter SLC2A1 (Shulman and Rothman, 2017). Unlike SLC5A8, SLC2A1 is highly expressed in most tumors.

GWAS analysis demonstrated that variants of SLC8A1 were closely related to the risk of colorectal neoplasia and calcium intake and inversely correlated with colorectal adenomas (Zhao et al., 2017a). SLC8A1 functions as a Na^+^/Ca^2+^ exchanger and mediates the extrusion of Ca^2+^ ion across the cell membrane (Khananshvili, 2013). Ca^2+^ signals from SLC8A1 participate in immunology and are required for inflammatory TNF-α production in intestine (Staiano et al., 2009). More importantly, SLC8A1 is essential for PKC-α activation, which further participates in ERK1/2 phosphorylation in endothelial cells (Andrikopoulos et al., 2011). Thus, tumor angiogenesis may be regulated by SLC8A1 expression.

There are some SLCs linked to cancer risk, although the substrates or mechanisms remain unclear. SLC39A6 has been associated with esophageal cancer suppression, and a GWAS showed that it may contribute to longer survival (Wu et al., 2013). Another GWAS has identified SLC13A2 as a risk factor for familial HBV-related hepatocellular carcinoma (Lin et al., 2017).

## SLCs in nutrient and metabolite sensing and regulation

In recent years, a great deal of GWAS of complex diseases have been published, and various risk loci have been successfully identified. However, the progress of exploring identified functional genes related to diseases has been relatively slow. Many of these loci were later required to be functionally confirmed by global or tissue-specific gene knockout experiments in mice (summarized in Table 2).

**Table 2:** Animal model phenotypes relative to representative Slc transporters.

Slc	Diseases/defects	Animal models	References
Slc2a4	Fasting hyperglycemia and glucose intolerance	Global Slc2a4-deficient mice	Katz et al. (1995)
Slc2a4	Glucose uptake reduced by 75%	Muscle-specific Glut4 knockout (KO) mice	Zisman et al. (2000)
Slc2a4	Insulin resistance secondarily in muscle and liver	Adipose-specific Glut4 KO mice	Yang et al. (2005)
Slc2a4	Impaired ability in mouse under stress	Cardiac-specific Glut4 KO mice	Wende et al. (2017)
Slc2a4	Protection from albuminuria and diabetic nephropathy	Podocyte-specific Glut4 KO mice	Guzman et al. (2014)
Slc2a9	Hyperuricaemia, hyperuricosuria, spontaneous hypertension, dyslipidemia, and elevated body fat	Glut9 KO mice	DeBosch et al. (2014)
Slc3a1	Cystinuria	Slc3a1 KO Feline	Mizukami et al. (2015)
Slc5a2	Prevention from HFD-induced hyperglycemia and glucose intolerance; reduced plasma insulin concentrations	Slc5a2 KO mice	Jurczak et al. (2011)
Slc6a15	Less anxiety- and depressive-like behavior	Slc6a15 KO mice	Santarelli et al. (2016)
Slc6a15	Increased anxiety-like behavior	Slc6a15 overexpression mice	Santarelli et al. (2016)
Slc11a1	Sensitive to *Salmonella typhimurium* infection	Slc11a1 KO mice	Caron et al. (2006)
Slc12a1	Decreased neuronal layer thickness and cell number; more immature interneurons	Slc12a1 KO mice	Haering et al. (2015); Magalhaes and Rivera (2016)
Slc13a1	Hyposulfatemia	NaS1-null (Nas1^−/−^) mice	Markovich (2012b)
Slc15a1	Higher plasma amino acid levels	Slc15a1 KO mice	Yang et al. (2013)
Slc15a2	Lower body weight and lower relative heart weight in male PEPT2-null mice; lower relative kidney weight in female mice	PEPT2-null mice	Frey et al. (2006)
Slc16a1	Hyperinsulinism	RIP7-rtTA/Mct1-Luc mice	Pullen et al. (2012)
Slc17a1	Normal plasma Pi and calcium levels; reduced Pi excretion	NPT1^−/−^ mice	Miyamoto et al. (2011)
Slc19a3	Neurodegenerative disorder	Slc19a3-deficient mice	Suzuki et al. (2017)
Slc20a2	Dysregulated phosphate homeostasis basal ganglia calcification	Heterzygous (Het) Slc20a2 mice	Jensen et al. (2013)
Slc23a1	Lower plasma ascorbate concentrations; brain hemorrhage	Slc23a1 KO mice	Sotiriou et al. (2002)
Slc24a4	A deficit in olfactory neurons	Slc24a4 KO mice	Li and Lytton (2014)
Slc26a1	Hyposulfatemia, hyperoxalemia; transport anions including sulfate, bicarbonate, chloride, and oxalate	Sat1-null (Sat1^−/−^)/Sat1 KO mice	Markovich (2012a)
Slc27a1	Reduced insulin resistance; decreased electroretinogram response	Slc27a1 KO mice	Kim et al. (2004); Chekroud et al. (2012)
Slc30a8	Islets with markedly fewer dense cores but more rod-like crystals	ZnT8-null (Slc30a8^−/−^) mice	Nicolson et al. (2009)
Slc38a3	Stunted growth, altered amino acid levels, hypoglycemia, and 20-day life; higher glutamine but reduced glutamate and γ-aminobutyric acid (GABA) levels in brain; reduced renal ammonium excretion	Snat3 mutant mice; Snat3-deficient mice	Chan et al. (2016)
Slc39a14	Impairs hepatic Mn uptake and biliary excretion, resulting in the accumulation of Mn in the circulation and brain	Global Slc39a14 KO mice; hepatocyte-specific Slc39a14 KO mice	Xin et al. (2017)

Slc functions were identified in genetically modified animal models. Slc deficiency has the potential to cause direct metabolic disorders or increase the susceptibility to diseases.

### Amino acids, glucose, and lipids—major nutrients

Dietary carbohydrate is necessary for supplying humans with essential saccharides and energy. SLC2A4 (GLUT4) has well-established roles as a glucose transporter affecting the body glucose disposal rate in adipose, muscle, and cardiac tissues (Mueckler and Thorens, 2013). SLC2A4 global-deficient mice exhibited fasting hyperglycemia and glucose intolerance, while overexpression of SLC2A4 in adipose tissue resulted in alleviating insulin resistance (Yang et al., 2005). In a tissue-specific model, the muscle in mice lacking SLC2A4 displayed a loss of glucose uptake compared to wild-type, and cardiac deletion of SLC2A4 resulted in an impaired ability in stressed mice (Zisman et al., 2000; Wende et al., 2017). In recent years, it was found that podocyte-specific GLUT4-deficient mice did not develop albuminuria and diabetic nephropathy (Guzman et al., 2014). These results indicated that the main target tissues of SLC2A4 are adipose and muscle for metabolic disease.

Dietary proteins and their amino acid products are essential for the maintenance of human nutrition and development. SLC15A1 regulates the absorption and homeostasis of most amino acids in the intestine (Daniel, 2004). Furthermore, almost all the plasma amino acid levels of SLC15A1-deficient mice were significantly increased compared to wild-type (Nassl et al., 2011). Interestingly, the intestinal amino acid absorption regulated by SLC15A1 is notable only after high dietary protein intake (Nassl et al., 2011). Considering all these, SLC15A1 has contributed to amino acids absorption from intestine and transport from circulation to whole body. SLC15A2, mainly expressed in the kidney, prevents the urinary loss of amino acids and assists with meeting the nutritional needs of the body by renal reabsorption of these amino acids and peptides (Nassl et al., 2011). Furthermore, SLC15A2^−/−^ animals possessed lower body weight and relative heart weight compared with wild-type animals, suggesting a loss of amino acids in the heart (Frey et al., 2006). SLC15A2 controls amino acids transport from tissue to circulation which differs from SLC15A1.

Glutamine, mainly transported by SLC38A3, is the most abundant amino acid in the body and is involved in various processes (Curi et al., 2005). SLC38A3 expression linked with tissue development is demonstrated by short life of SLC38A3 mutant mice with hypoglycemia, suggesting that glutamine transport is crucial for growth and development (Chan et al., 2016). Additionally, the urea levels and renal ammonium excretion were decreased in SLC38A3-deficient mice (Chan et al., 2016). To sum up, SLC38A3 influences the glutamine accumulation in tissues and interacts with other amino acids.

MTORC1, an important amino acid-sensing molecule that regulates cell growth, has been considered as a key cancer signaling pathway (Guertin and Sabatini, 2007). SLC38A9 has been recently demonstrated to transport amino acids and regulate the downstream of mTORC1 signaling in lysosomes, which provides higher levels of amino acids for the cell (Wyant et al., 2017). Arginine is one of the elusive sensors for the mTORC1 pathway. SLC38A9 exhibits low amino acid-sensing activity for arginine transport, which suppresses mTORC1 activity (Wolfson and Sabatini, 2017). As a high-affinity transporter for leucine, the amino acid preference of SLC38A9 is leucine. In an animal feeding study, the Slc38a9 expression in the hypothalamus of the brain was upregulated by a high-fat diet, suggesting that Slc38a9 has an impact on eating behavior (Hellsten et al., 2017). It is predicted that SLC38A9 may regulate cancer signaling and eating behavior via leucine.

As a major nutrient, fatty acids play an important physiological role in the regulation of body weight, energy metabolism, insulin sensitivity, cell-surface receptors, and brain development (Das, 2006). The accumulation of lipids and the transport of fatty acids among tissues lead to various obesity-related metabolic diseases.

SLC27A1, one of the fatty acid transport proteins, is highly expressed in skeletal muscle, heart, and adipose tissue for long-chain fatty acid uptake (Anderson and Stahl, 2013). SLC27A1 deficiency has a limited impact on the alleviation of whole-body adiposity, but protects high-fat-fed mice from insulin resistance (Kim et al., 2004). SLC27A1 has been implicated in significantly reducing large cardiac lesion areas in Ldlr^−/−^ mice (Zhao et al., 2017b). Moreover, SLC27A1 knockout mice displayed a decrease in the neuroretina and response to light, leading to an aging process (Chekroud et al., 2012). In addition, SLC27A1 was recently found to enhance the transport of docosahexaenoic acid (DHA) into the brain via the membrane of brain microvessel endothelial cells (Ochiai et al., 2017). Considering all these, SLC27A1 may confer an anti-inflammation role in tissues which influence the process of inflammation-induced injury or aging.

### Vitamins, metallic trace elements—trace nutrients

Ascorbic acid (vitamin C) is essential for its antioxidant role in the body (Padayatty et al., 2003). A deficiency of SLC23A1 in mice causes respiratory failure and intracerebral hemorrhage in newborn mice, implying that SLC23A1 is essential for lung alveolar expansion and blood vessel development in the brain (Padayatty et al., 2003). Human polymorphisms in SLC23A1 have also been associated with aggressive periodontitis and Crohn’s disease (Amir Shaghaghi et al., 2014; de Jong et al., 2014). These results have proved that SLC23A1 has a deep impact on tissue development beyond antioxidant vitamin C uptake.

Vitamin B1, mediated by Slc19a3 transport, is an important nutrient for energy production, and its deficiency has been linked to cardiac failure, neurological disorders, and oxidative stress (Collie et al., 2017). In Slc19a3-deficient mice, the animals displayed a neurodegenerative disorder, and a high dose of thiamine intake alleviated neurodegeneration (Suzuki et al., 2017). Neurological damage also was demonstrated in humans with inherited thiamine defects (Ortigoza-Escobar et al., 2017). Thus, SLC19A3 is mainly contributed to brain thiamine transport.

Iron is a crucial cofactor for important biological processes including cellular survival, cell death, respiration reduction reaction, and transport of oxygen via hemoglobin (Lawen and Lane, 2013). SLC11A1 has been shown to transport iron. The SLC11A1 knockout or transgenic model is highly susceptible to diarrhea and colitis caused by *Salmonella* infection by inducing an inflammatory response and macrophage recruitment. Thus, SLC11A1 plays a critical role in host defense against infectious *Salmonella* (Woo et al., 2008; Valdez et al., 2009).

SLC39A8 (ZIP8) contributes to the uptake of zinc into host cells, thereby providing the competitive advantage of zinc influx. SLC39A8 hypomorphic mice exhibited dysregulated zinc uptake and increased NF-κB activation (Liu et al., 2013). The mechanism of SLC39A8 in the host defense that negatively regulates NF-κB through IKK-β signaling might potentially protect against infection.

SLC11A1, SLC39A8, and SLC30A10 were not only implicated in iron or zinc transport but they also influenced Mn distribution in the body (Tuschl et al., 2013). Moreover, it was found that global loss of the Slc39a14 gene increases brain Mn accumulation, leading to motor deficits in the mouse (Xin et al., 2017). Additionally, it was found that Slc39a14 liver-specific-deficient mice had no motor deficits due to no increase in Mn levels in brain tissues, indicating that liver Slc39a14 expression has no effect on whole-body Mn homeostasis. To sum up, Mn transport is influenced by other metal ions transporters and Slc39a14 is the main transporter in brain.

### Metabolite transport and interaction with microbiota

Despite the presence of nutrients, many metabolites are also transported or influenced by SLCs. Intestinal serotonin, produced by tryptophan metabolism from enterochromaffin cells, is implicated in stimulating the nervous system and GI function (Bhattarai et al., 2017). SLC6A4, the main serotonin transporter in the intestine, was influenced by gut microbiota composition (Bhattarai et al., 2017). The germ-free mice displayed increased colonic SLC6A4 expression and reduced serotonin levels compared to normal commensal microbiota mice. Furthermore, SLC6A4 knockout mice exhibited decreased gut serotonin levels, indicating that a serotonin-deficient compensatory response regulated by SLC6A4 relied on the microbiota (Yano et al., 2015). Thus, SLC6A4 regulates serotonin distribution via brain–gut axis.

Dietary fiber also contributes to human health, and low fiber intake is associated with obesity-related metabolic disease (Fernandez-Navarro et al., 2017). An important mechanism involved is that short-chain fatty acids, produced by the intestinal fermentation of dietary fiber, can modulate the microbiota, reduce the appetite, and protect against chronic inflammation (Correa-Oliveira et al., 2016). SLC16A1, which is a butyric acid transporter, maintains luminal butyrate levels and thus provides fuel for mucosal cells (Jones and Morris, 2016). In contrast, SLC16A1 expression was downregulated in inflammatory tissues with lower butyric acid levels in the gut (Thibault et al., 2007). Moreover, β-cell-specific SLC16A1 overexpression in transgenic mice exhibited decreased fasting blood glucose levels (Pullen et al., 2012). It is concluded that gut and pancreatic butyric acid transport mediated by SLC16A1 to reduce chronic inflammation.

## SLCs for drug targets

There has been increased interest in the targets of SLCs for drug development. A review of molecular drug targets found that 12 SLCs out of 435 human genes could be used as drug targets (Rask-Andersen et al., 2011). Because most SLCs contribute to the transport of small organic molecules, it is predicted that the drug development of SLCs will be promising.

There are seven classes of SLCs that have been approved for market and nine classes of SLC drugs in clinical trials (Figure 3B and Supplementary Figure S1) according to two reviews of previous research (Rask-Andersen et al., 2013; Cesar-Razquin et al., 2015). These drug targets are linked to a wide variety of diseases such as hyperglycemia, depression, addiction, uricosuria, gout, and cancer. Among these drug targets, the SLC5 and SLC6 classes have been the most intensively studied targets. Dapagliflozin, the most well-known SGLT2 (sodium-glucose cotransporter 2 or SLC5A2) inhibitor, has been reported to attenuate blood glucose concentration in an insulin-independent manner. Clinical trials revealed that dapagliflozin safely reduced hyperglycemia without electrolyte disturbances, hepatotoxicity, or nephrotoxicity (Musso et al., 2012). Fluoxetine, the most utilized SLC6A4 inhibitor, could benefit adolescents with major depressive disorder by decreasing suicidal thinking (March et al., 2004).

Another important drug target for neurological disorders is the SLC class. NKCC1 has a favorable chloride transport capacity to accumulate intracellular Cl^−^ levels in immature neurons and interact with K^+^–Cl^−^ cotransporter (KCC, SLC12A5) (Jaggi et al., 2015). An NKCC1 deficiency results in a reduction of immature interneuron migration and a decrease of ~20% of the neuronal layer thickness, suggesting that NKCC1 contributes to the dynamic equilibrium of neurogenesis in mice (Haering et al., 2015; Magalhaes and Rivera, 2016). Selective NKCC1 inhibitors known as diuretics including bumetanide and furosemide improve neurological behavior control and lower anxiety, neuropathic pain, and schizophrenia in animals and patients (Ben-Ari, 2017).

## Perspective

Compared to other gene families, SLCs are not well established, which is why this field is promising. In recent years, CRISPER/Cas9 has been a gene editing tool with high efficiency, and a great number of SLC knockout animal models were constructed for screening risk loci and drug targets of diseases. Moreover, there also remains a great need for two or more SLC gene knockout animals to uncover the interaction of SLCs, especially those that share the same transporting substrates.

We still face challenges in understanding the SLC contribution to human health. It is very likely that a large number of substrates for SLCs have not yet been identified, and the major substrates are also unknown. An important fact is that many SLC risk loci of human diseases have not been verified in gene-deficient animal models. This has occurred partly because of different tissue expression of target genes in different mammalian species. Other interesting fields may involve different diseases that share the same SLC risk loci. For example, SLC22A23 is not only a locus for Crohn’s disease but also a risk gene for bronchodilator responsiveness in asthma (Table 1). Another SLC6A13 locus contributes to both elevated blood pressure and CKD (Table 1). Thus, the exploration of SLCs may give insight into complex human diseases.

SLC transporters have aroused the attention of the pharmaceutical industry. Most recently, Jnana Therapeutics Inc., the first medical company focusing on SLC transporters, was established in Boston, MA, USA and received $50 million Series A financing (https://www.jnanatx.com/). The aim of this company is to explore the mechanism of SLC-associated immunometabolism, lysosomal function, and mucosal defense and develop drugs for immuno-oncology, inflammatory disorders, and neurological diseases.

In conclusion, a global understanding of SLC transporters will guide novel nutritional strategies, promote metabolic assessment, and facilitate drug development.

## Supplementary Material

Supplementary Data

Supplementary Data

Supplementary Data
