# Supplementary material for: The SLC transporter in nutrient and metabolic sensing, regulation, and drug development
Source: J Mol Cell Biol. 2018 Sep 18;11(1):1–13. doi: 10.1093/jmcb/mjy052 (PMC6359923; doi:10.1093/jmcb/mjy052)
Supplement: Supplementary Data [file mjy052_supplementary_material.pdf]

Supplementary Methods

Construction of the phylogenetic tree for human SLCs

A total of 375 members' sequence information was obtained from the SLCs database (http://slc.bioparadigms.org/) and further the reviewed protein were screened out from the UniProt database(http://www.uniprot.org/).According to hmm (hidden Markov models, HMM) profile from Pfam database (http://pfam.xfam.org/), the hmmscan programme was applied to process the data from functional domains of SLCs as previously described(Michel et al., 2017).

Software QuickProbs 2 was used for sequence alignment since SLCs contain plenty of family members (Gudys and Deorowicz, 2017).And then, TrimAl software was applied to process the aligned data and the columns with surplus gaps were removed when the cutoff value was set at -gt 0.8 (Capella-Gutierrez et al., 2009). See from the file“SLC_trimal.clw”. Finally, phylogenetic tree was constructed by maximum likelihood of FastTree software with modified parameters according to literature(Price et al., 2010;Xiong et al., 2015). See from the file “SLC_fastTree.nwk”.

Supplementary References

Capella-Gutierrez, S., Silla-Martinez, J.M., and Gabaldon, T. (2009). trimAl: a tool for automated alignment trimming in large-scale phylogenetic analyses. Bioinformatics 25, 1972-1973.

Gudys, A., and Deorowicz, S. (2017). QuickProbs 2: Towards rapid construction of high-quality alignments of large protein families. Sci. Rep. 7, 41553.

Michel, M., Skwark, M.J., Menendez Hurtado, D., et al. (2017). Predicting accurate contacts in thousands of Pfam domain families using PconsC3. Bioinformatics *33*, 2859-2866.

Price, M.N., Dehal, P.S., and Arkin, A.P. (2010). FastTree 2--approximately maximum-likelihood trees for large alignments. PLoS One 5, e9490.

Xiong, J., Feng, J., Yuan, D., et al.(2015). Tracing the structural evolution of eukaryotic ATP binding cassette transporter superfamily. Sci. Rep. 5, 16724.

Supplementary Table S1

Representative SLCs transporter-mediated drug transport . SLCs play a crucial role in uptake and efflux of drugs. The scope of SLC drug transporters exert a wide range including hypoglycemic drugs (Metformin and Dapagliflozin), hypolipidemic drugs (Simvastatin), anti-aids drug(Tenofovir), diuretic drugs (Spironolactone and Torasemide), antihypertensive drug (Sacubitril, Enalapril and Telmisartan), anti-cardiovascular diseases drug (Atenolol) and even chemotherapeutic agents (Fluorouracil, Mitoxantrone, Methotrexate, Gemcitabine, Cisplatin and Oxaliplatin).

SLCs	Drugs	Chemical structure
SLC22A1<br>SLC22A2<br>SLC22A3	Metformin	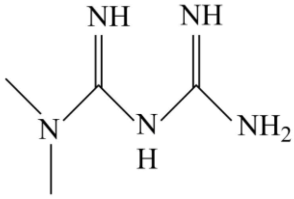
SLC22A1<br>SLC22A2<br>SLC22A3	Cisplatin/<br>Oxaliplatin	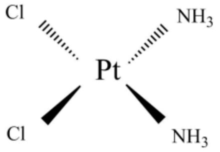 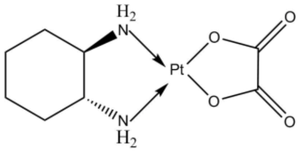
SLC22A4	Mitoxantrone	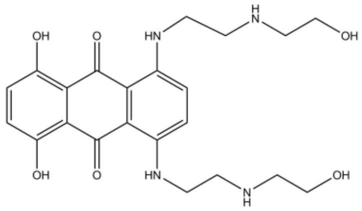
SLC22A5	Spironolactone	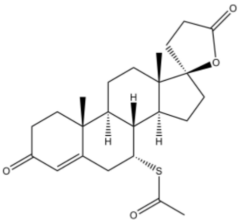
SLC22A6	Tenofovir	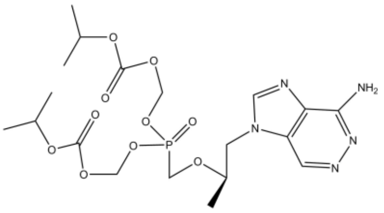
SLC22A7	Fluorouracil	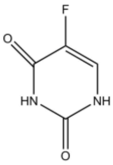
SLC22A8	Methotrexate	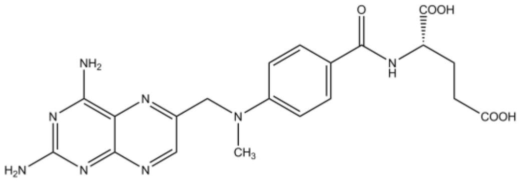
SLC22A11	Torasemide	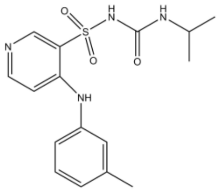

SLCO1A1	Enalapril	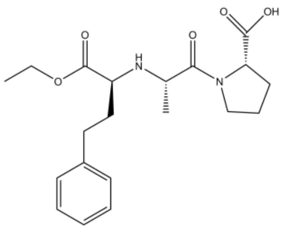
SLCO1B1	Simvastatin	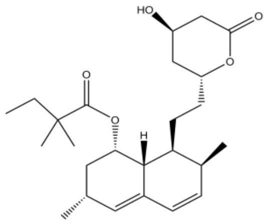
SLCO1B3	Sacubitril	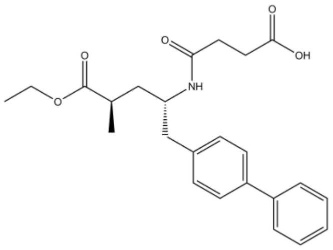
SLCO1B3	Telmisartan	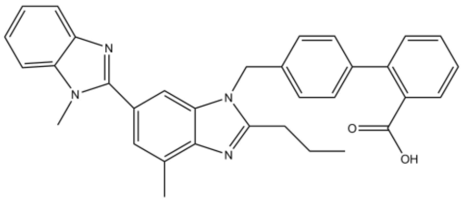
SLC28A3	Gemcitabine	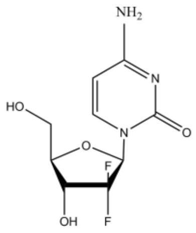
SLC47A1<br>SLC47A2	Atenolol	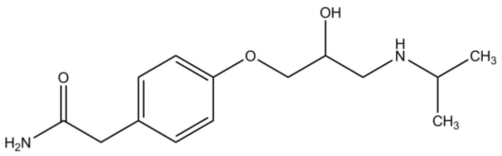

Supplementary Figure S1 Drugs of SLCs inhibitors and their chemical structure targeting SLCs. Data was cited from Rask-Andersen et al. (2013) and Cesar-Razquin et al. (2015).

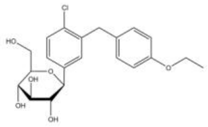
Dapagliflozin(SLC5A2)

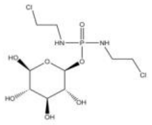
Glufosfamide(SLC2A1)

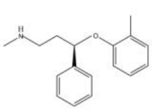
Atomoxetine (SLC6A2)

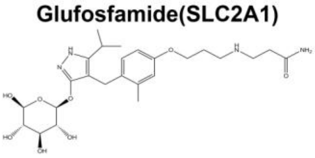
KGA-2727(SLC5A1)

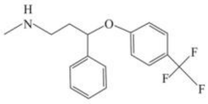
Fluoxetine(SLC6A4)

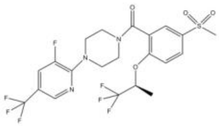
Bitopertin（SLC6A9）

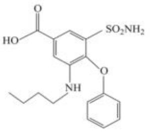
Bumetanide(SLC12A1)

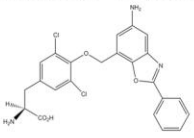
JPH203(SLC7A5)

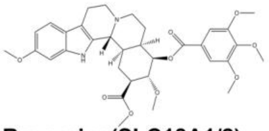
Reserpine(SLC18A1/2)

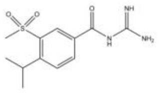
Cariporide (SLC9A1)

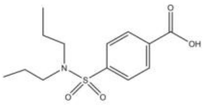
Probenecid(SLC22A6)

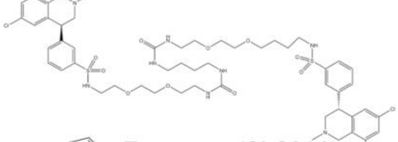
Tenapanor(SLC9A3)

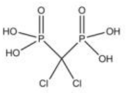
Clodronate(SLC25A4/5/6)

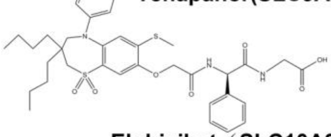
Elobixibat（SLC10A2）

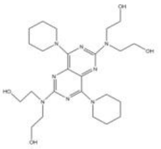
Dipyridamole (SLC29A1)

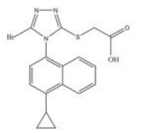
Lesinurad (SLC22A12)
